# Blood pressure and falls in community-dwelling people aged 60 years and older in the VHM&PP cohort

**DOI:** 10.1186/1471-2318-13-50

**Published:** 2013-05-21

**Authors:** Diana Klein, Gabriele Nagel, Andrea Kleiner, Hanno Ulmer, Barbara Rehberger, Hans Concin, Kilian Rapp

**Affiliations:** 1 Department of Clinical Gerontology, Robert-Bosch-Hospital, Stuttgart, Germany; 2 Institute of Epidemiology and Medical Biometry, Ulm University, Ulm, Germany; 3 Agency for Social- and Preventive Medicine, Bregenz, Austria; 4 Department of Medical Statistics, Informatics and Health Economics, Innsbruck Medical University, Innsbruck, Austria

**Keywords:** Falls, Blood pressure, Hypotension, Risk factors

## Abstract

**Background:**

Falls are one of the major health problems in old people. Different risk factors were identified but only few epidemiological studies analysed the influence of conventionally measured blood pressure on falls. The objective of our study was to investigate the relationship between systolic and diastolic blood pressure and falls.

**Methods:**

In 3,544 community-dwelling Austrian women and men aged 60 years and older, data on falls within the previous three months were collected by questionnaire. Blood pressure was measured by general practitioners within the Vorarlberg Health Monitoring and Prevention Programme (VHM&PP) 90 to 1095 days before the fall assessment. A multiple logistic regression analysis was conducted. The models were stratified by gender and adjusted by age, number of medical conditions and subjective feeling of illness.

**Results:**

In total, 257 falls in 3,544 persons were reported. In women, high systolic and diastolic blood pressure was associated with a decreased risk of falls. An increase of systolic blood pressure by 10 mmHg and of diastolic blood pressure by 5 mmHg reduced the risk of falling by 9% (OR 0.91, 95% Cl 0.84-0.98) and 8% (OR 0.92, 95% Cl 0.85-0.99), respectively. In men, an increased risk of falls was observed in participants with low systolic or low diastolic blood pressure.

**Conclusions:**

Blood pressure was associated with the risk of falls. Hypertensive values decreased the risk in women and low blood pressure increased the risk in men.

## Background

Falls are one of the major health problems in old people. About one third of people aged 65 years and older report at least one fall per year [1]. Consequences can be injuries such as fractures of the hip, the humerus or the forearm, fear of falling, loss of independence and increased mortality [2-4]. Different risk factors for falls including functional limitations and several diseases have been identified [5]. Cardiovascular diseases, for example, have been found to be associated with falls, mostly as a result of hypotensive episodes [6,7]. Blood pressure (BP) is one of the leading risk factors for cardiovascular diseases. Increasing BP has been shown to be linearly associated with cardiovascular disease and mortality. Therefore, low BP values have been usually regarded as a protective factor for different diseases and death [8,9].

In old people, however, there is evidence that the positive relationship between BP and mortality is weakened and that a low BP may even increase mortality [10-12]. For example, in the INVEST-study a J-curve between BP and adverse outcomes (all-cause mortality, nonfatal myocardial infarction or nonfatal stroke) was observed with an increased risk in participants with a very low and a very high BP [13].

Orthostatic hypotension has been shown to be a risk factor for recurrent falls in nursing home residents [14] and in people living in apartments for the elderly [15]. However, assessing orthostatic hypotension is relatively time-consuming and Schellong tests or tilt table testing are rarely performed in daily practice. In contrast, simple BP measurements are one of the basic examinations and available for nearly every patient. It is therefore surprising that only few epidemiological studies exist which analysed the influence of conventionally measured BP on falls.

The objective of our study was to investigate the relationship between BP and falls in more than 3,500 community-dwelling people aged 60 years and older.

## Methods

### Study design and study population

Data from the Austrian Vorarlberg Health Monitoring and Prevention Programme (VHM&PP) were analysed. In Vorarlberg, the westernmost province of Austria, medical examinations are routinely performed by general practitioners within the VHM&PP. All Austrian citizens aged 19 years and older can voluntarily participate in these examinations once a year. The participation must be arranged on ones own initiative and costs are covered by the participant’s health insurance. The medical examinations include amongst others, the measurement of BP. The VHM&PP has been described in detail previously [16].

Between 2000 and 2004 patients aged 60 years and older were asked to complete an additional questionnaire on age-related health which included questions about their current health status, living conditions, their functional impairments or falls. The questionnaire was handed out before or directly at the health examination and completed at home. All participants gave their written informed consent. For this study, institutional review board approval was obtained by the Ethics Committee of the province of Vorarlberg.

The current analysis was restricted to participants (N=3,544) who had a health examination 90 to 1095 days prior to completing the questionnaire. If participants attended more than one health examination during this period, the BP measurements of the latest examination was matched to the information of the questionnaire. A minimum of 90 days was chosen in order to enable a prospective analysis since falls were assessed retrospectively over a period of three months (see below). BP is a dynamic value which may change over time. Therefore, a maximum of 1095 days (i.e. three years) was chosen to increase the propensity that the exposition was actually associated with the outcome.

### Outcome

The outcome variable of the study was a fall. Fallers were defined as those who reported one or more falls during the last three months. This was assessed by the questionnaire: “Did you fall in the previous three months?”

### Blood pressure

Systolic (SBP) and diastolic (DBP) blood pressure were measured at the medical examination in sitting position with a mercury sphygmomanometer. SBP and DBP were analysed as continuous, dichotomous and categorical variables. The dichotomous variable differentiated BP values regarded as normotensive or hypertensive (<140 and ≥140 mmHg for SBP and <90 and ≥90 mmHg for DBP) [8]. For the categorical variable SBP was divided in intervals of 20 mmHg (<120, 120-<140, 140-<160, 160-<180, ≥180) and DBP in intervals of 10 mmHg (<80, 80-<90, 90-<100, ≥100). The normotensive intervals (120-<140 mmHg for SBP and 80-<90 mmHg for DBP) were used as reference categories. In men, the two highest categories were combined to guarantee at least 10 falls in each category. The mean arterial pressure (MAP) was calculated as DBP+1/3(SBP-DBP) and included in the model only as a continuous variable.

### Covariates

In order to adjust the model for co-morbidity, number of medical conditions and subjective feeling of illness were included. Medical conditions were assessed in the questionnaire by the following questions: ‘Are you or have you been under medical treatment because of (1) high blood pressure (hypertension), (2) other cardiovascular diseases or stroke, (3) pulmonary diseases, (4) diabetes mellitus, (5) cancer, (6) chronic urinary tract infection or (7) other diseases’. Through addition of the positive answers of the single items an additive co-morbidity score was built (range 0–7) and included in the model as continuous variable. Subjective feeling of illness was measured by asking ‘Do you currently feel sick?’ (yes/no). Self-rated health has been shown to be a good independent predictor of morbidity and all-cause mortality [17,18]. Therefore, subjective feeling of illness was included as a second covariate for the participant’s health status in the fully adjusted model.

Information about the use of BP medication was only based on the question if the participants are or have been under medical treatment for hypertension and was collected at the end of the study. The questionnaire did not provide information if the participants actually used BP medication at the time of the BP measurement. Therefore, information about the use of antihypertensives at baseline may be inaccurate.

### Statistical analysis

A multiple logistic regression analysis was conducted to calculate odds ratio (OR) of falls. BP was included into the models as continuous, dichotomous and categorical variable. Presented are associations adjusted for age (model 1) and for age, subjective feeling of illness and number of medical conditions (model 2). All analyses were stratified by gender.

Furthermore, in three different supplementary analyses model 2 was either adjusted for antihypertensive treatment, or stratified by age (the median of age served as cut-point) or by time period between BP measurement and outcome (the median of days served as cut-point). Effect modification between SBP or DBP and antihypertensive treatment was analysed by including an interaction term in the model.

## Results

The study consisted of 1,970 women and 1,574 men with a median age of 70 and 69 years, respectively. About 9% of the women and 5% of the men reported at least one fall within the three months before completing the questionnaire. A hypertensive SBP (≥ 140 mmHg) was measured in 70.5% of the female and 67.6% of the male study population. The proportion of women and men with a DBP defining hypertension (≥ 90 mmHg) was considerably lower. A subjective feeling of illness was reported by 17.2% of the women and 13.6% of the men (Table 1).

**Table 1:** Characteristics of the study population

	**Women**		**Men**	
	**N**	**Falls N (%)**	**N**	**Falls N (%)**
Participants (N)	1970	180 (9.1%)	1574	77 (4.9%)
Systolic blood pressure (mmHg)				
<120	87	10 (11.5%)	95	10 (10.5%)
120-139	495	51 (10.3%)	415	19 (4.6%)
140-159	825	73 (8.8%)	651	28 (4.3%)
160-179	389	33 (8.5%)	293	14 (4.8%)
≥180	174	13 (7.5%)	120	6 (5.0%)
Diastolic blood pressure (mmHg)				
<80	386	38 (9.8%)	316	23 (7.3%)
80-89	955	100 (10.5%)	816	34 (4.2%)
90-99	434	31 (7.1%)	330	15 (4.5%)
≥100	195	11 (5.6%)	112	5 (4.5%)
Age, median (range), (years)	70 (60–97)		69 (60–91)	
Number of medical conditions^*^, median (range)	1 (0–7)		1 (0–6)	
Subjective feeling of illness^†^, N (%)	338 (17.2%)		214 (13.6%)	

* Assessed medical conditions: high blood pressure (hypertension); other cardiovascular diseases or stroke; pulmonary diseases; diabetes mellitus; cancer; chronic urinary tract infection; other diseases.

^†^ Assessed by the question ‘Do you currently feel sick?’ (yes/no).

In women, increasing blood pressure values were associated with a decreased risk of falls, with hypertensives showing a decreased risk compared with normotensives, and the protective effect being stronger for DBP. In the fully adjusted model an increase of SBP by 10 mmHg and of DBP by 5 mmHg reduced the risk of a fall by 9% (Odds ratio (OR) 0.91, 95% confidence interval (Cl) 0.84-0.98) and 8% (OR 0.92, 95% Cl 0.85-0.99), respectively. Women with a DBP of at least 100 mmHg, for example, had only half the fall risk compared to women with a DBP between 80 and 90 mmHg. For increasing categorical BP values decreasing risk estimates were observed. In contrast to DBP estimates for SBP did not reach statistical significance (Table 2).

**Table 2:** Influence of systolic, diastolic and mean arterial blood pressure on falls in women and men aged 60 years and older in the VHM&PP cohort

	**Women**		**Men**	
	**Odds ratio (95% confidence interval)**		**Odds ratio (95% confidence interval)**	
	Model 1^*^	**Model 2**^**†**^	Model 1^*^	**Model 2**^**†**^
**Systolic blood pressure**				
Increase of 10 mmHg	0.92 (0.85-1.00)	0.91 (0.84-0.98)	0.92 (0.81-1.03)	0.91 (0.81-1.03)
Dichotomous (mmHg)				
<140	1.00	1.00	1.00	1.00
≥140	0.76 (0.55-1.06)	0.71 (0.51-0.99)	0.73 (0.45-1.18)	0.71 (0.44-1.16)
Categorical (mmHg)				
<120	1.19 (0.58-2.45)	1.09 (0.52-2.26)	2.55 (1.14-5.71)	2.46 (1.10-5.54)
120-<140	1.00	1.00	1.00	1.00
140-<160	0.83 (0.57-1.21)	0.76 (0.52-1.11)	0.90 (0.49-1.64)	0.87 (0.48-1.59)
160-<180	0.75 (0.47-1.19)	0.68 (0.42-1.09)	0.96 (0.50-1.84) ^‡^	0.93 (0.48-1.80) ^‡^
≥180	0.67 (0.35-1.26)	0.60 (0.32-1.15)		
**Diastolic blood pressure**				
Increase of 5 mmHg	0.91 (0.84-0.99)	0.92 (0.85-0.99)	0.93 (0.82-1.05)	0.93 (0.82-1.05)
Dichotomous (mmHg)				
<90	1.00	1.00	1.00	1.00
≥90	0.63 (0.44-0.90)	0.62 (0.43-0.89)	0.90 (0.53-1.51)	0.90 (0.53-1.51)
Categorical (mmHg)				
<80	0.93 (0.62-1.38)	0.90 (0.61-1.35)	1.76 (1.02-3.05)	1.77 (1.02-3.07)
80-<90	1.00	1.00	1.00	1.00
90-<100	0.66 (0.43-1.00)	0.65 (0.42-0.99)	1.08 (0.62-1.91) ^#^	1.09 (0.62-1.91)^#^
≥100	0.52 (0.27-0.99)	0.50 (0.26-0.96)		
**Mean arterial pressure**				
Increase of 10 mmHg	0.84 (0.74-0.97)	0.83 (0.73-0.96)	0.86 (0.70-1.05)	0.85 (0.69-1.05)

* Model 1: adjusted for age.

† Model 2: adjusted for age, subjective feeling of illness and number of medical conditions.

‡ Systolic blood pressure ≥160 mmHg.

# Diastolic blood pressure ≥90 mmHg.

Men showed a clearly increased risk for low SBP (<120 mmHg) and DBP (<80 mmHg) with a 2.5 times and 1.8 times higher risk of falling than those with ‘high normal’ values, respectively. No significant association between BP as a continuous or a dichotomous variable and falls was found (Table 2).

In the analysis stratified by age, the association between SBP and falls were only observed in women and men over 70 years. In contrast, an association between DBP and falls was found in women in the younger age-group and in men in the older age-group (Additional file 1: Table SA).

An additional analysis was performed stratified by the time period between BP measurement and outcome. In men, a stronger association was observed when the time period between BP measurement and outcome was closer. In women, the time period had no considerable effect on the estimates (Additional file 2: Table SB).

The additional adjustment of the model by ‘antihypertensive treatment’ did not change the estimates considerably and no significant effect modification between SBP or DBP and antihypertensive treatment was observed (Additional file 3: Table SC).

## Discussion

We observed a decreased risk of falls in women with hypertensive values for SBP and DBP and an increased risk of falls in men with low SBP or DBP. In men, the association between BP<120/80 mmHg and falls was limited to participants over 70 years. Therefore, a BP which is regarded as optimal in a cardiovascular point of view may actually be risky concerning falls in the oldest men. The cause of the gender difference remained unclear and requires replication in further studies.

There are only few epidemiological studies which analysed the association between conventionally measured BP and falls. Kario [19] identified lower SBP as an independent predictor of falls in relatively healthy community-dwelling older people. The association was stronger in women than in men. In this study, however, BP was assessed in supine and standing position. Participants with a standing SBP below 140 mmHg had a 2.8 times increased risk of falls compared to people from the reference group (≥140 mmHg). DBP was not related to falls. In our study low SBP was shown to be a significant predictor of falls only in men. In women, higher values of DBP were associated with a decreased risk of falling.

Two meta-analyses of observational studies found both a slight to moderate increased risk of falls with antihypertensive treatment [20,21] and van der Velde and colleagues demonstrated a significant reduction of falls after withdrawal of cardiovascular medications [22]. On the other hand, the randomised controlled HYVET study demonstrated convincingly that the treatment of old and very old people with BP values of 160/90 mmHg and more reduced their cardiovascular and total mortality. The target BP for patients aged 80 years and more was 150/80 mmHg and it remained unclear if a further reduction of BP is beneficial [23]. Like in all other medication phase III trials falls were not assessed as an additional adverse outcome in old people. In our analysis there was no effect modification between BP and antihypertensive treatment and adjustment for antihypertensive treatment did not change the results considerably. This is consistent with the study from Kario [19]. However, our data quality regarding the use of antihypertensive drugs was low and the results should therefore be interpreted with caution.

Several studies analysed the effect of orthostatic hypotension on falls. Orthostatic hypotension is defined as a decline of SBP or DBP of at least 20 or 10 mmHg within three minutes after transfer from sitting to standing, respectively. In some studies, orthostatic hypotension seemed to be a cause of (pre)syncopal episodes and falls in adults over 65 years of age [24-26]. Ooi et al., for example, identified orthostatic hypotension as an independent risk factor for recurrent falls in residents of nursing homes [14]. In contrast, another study in residents of nursing homes did not find orthostatic hypotension to be associated with falls [27]. In a study in community-dwelling people, orthostatic hypotension was most pronounced in patients with uncontrolled hypertension. In these patients the risk of falls was 2.5 times higher than in patients with uncontrolled hypertension but without an orthostatic hypotensive reaction. However, if all study participants independent of their baseline BP were included in the analysis, no association was found between orthostatic hypotension and falls [6].

The pathophysiological mechanism of an increased risk of falls in people with low BP or orthostatic hypotension may be partly due to changes in arterial structure and function such as vascular stiffness, calcification, collagen deposition and less distensibility of vessels. This may impair auto-regulation of BP and cause orthostatic hypotension and falls [28]. Our results suggest, whereby orthostatic dysregulation is not taken into account, that a lower baseline BP may imply an insufficient cerebral blood supply after a transfer to a standing position or whilst standing or walking which may be a hypothetical mechanism linking low BP with an increased fall risk. Recently higher systolic blood pressure has been shown to be associated with a lower annual increase in disability in the activities of daily living (ADL) and a lower annual decline in cognition [29]. Both variables are known to be associated with fall risk [5]. Disability in ADLs or cognition could be therefore mediating factors between BP and falls.

The strengths of the study are the longitudinal design and the large number of subjects. BP was measured in a standardized way by a health professional. The population of Vorarlberg is culturally and ethnically rather homogenous, with more than 90% of Austrian origin [16].

Our study has several limitations. Since there was no measurements in standing position it remains unclear if the observed effect was mediated by orthostatic dysregulation. Therefore, the pathophysiological mechanism remains hypothetical. Despite the large sample size, the number of participants and falls were low in some subgroups which reduced the statistical power of the analyses. Established risk factors for falls such as muscle weakness, imbalance, low walking speed [5] or the result of a cognition assessment, like the Mini Mental State Examination, were not available and therefore not adjusted for. However, the adjustment for co-morbidity (number of medical conditions) and subjective feeling of illness may have compensated to some extent the lack of further risk factors. In addition, we adjusted for the subjective reporting of ‘forgetfulness’ (data not shown), which did not change the estimates considerably. But even though we adjusted for co-morbidity residual confounding may have occurred. In addition, an underlying disease like heart failure, for example, may have been associated both with hypotension and falls. The study has a longitudinal nature since BP was measured before falls occurred. Falls, however, were assessed retrospectively by a questionnaire and could have therefore been subject for recall bias. Furthermore, the assessment of falls did not allow discriminating single fallers from frequent fallers.

The participants of the present study were probably in a relatively good health status since the preconditions to participate included the ability of a visit at the practice of the general practitioner in order to take part in the medical health examination and a fairly good cognitive state in order to complete the questionnaire. Furthermore, the initiative for participating in the examination was entirely left to the individuals. Thus, persons with mobility restrictions, care need, or impaired cognition like institutionalised people may have been underrepresented. This limits the external validity of our results, particularly since orthostatic BP dysregulation and falls are highly prevalent in very frail people [14,30].

## Conclusions

There is virtually no study which analysed the association between conventionally measured BP and falls. In the present study, high SBP and, more clearly, DBP were associated with a decreased risk of falls in women. In men low SBP or DBP were associated with an increased risk of falls. In daily practice BP is measured in most patients and patients with hypotension are easily recognisable. When treating them their fall risk should also be considered.

## Competing interests

The authors declare that they have no competing interests.

## Authors’ contributions

DK contributed by conception of the evaluation, analysis and interpretation of data and drafting the article. GN contributed by interpretation of data and drafting the article. AK contributed by analysis and interpretation of data. HU contributed by interpretation of data and drafting the article. BR contributed by conception of the study, interpretation of data and drafting the article. HC contributed by conception of the study, interpretation of data and drafting the article. KR contributed by conception of the evaluation, analysis and interpretation of data and drafting the article. All authors read and approved the final manuscript.

## Pre-publication history

The pre-publication history for this paper can be accessed here:

http://www.biomedcentral.com/1471-2318/13/50/prepub

## Supplementary Material

Additional file 1: Table SA Influence of systolic, diastolic and mean arterial blood pressure on falls in women and men aged 60 years and older stratified by age in the VHM&PP cohort. Click here for file

Additional file 2: Table SB Influence of systolic, diastolic and mean arterial blood pressure on falls in women and men aged 60 years and older stratified by the time period between BP measurement and outcome in the VHM&PP cohort. Click here for file

Additional file 3: Table SC Influence of systolic, diastolic and mean arterial blood pressure on falls additionally adjusted for BP treatment in women and men aged 60 years and older in the VHM&PP cohort. Click here for file
