# Supplementary material for: Blood pressure and falls in community-dwelling people aged 60 years and older in the VHM&PP cohort
Source: BMC Geriatr. 2013 May 21;13:50. doi: 10.1186/1471-2318-13-50 (PMC3663706; doi:10.1186/1471-2318-13-50)
Supplement: Additional file 1: Table SA — Influence of systolic, diastolic and mean arterial blood pressure on falls in women and men aged 60 years and older stratified by age in the VHM&PP cohort. [file 1471-2318-13-50-S1.pdf]

Additional Table A: Influence of systolic, diastolic and mean arterial blood pressure on falls in women and men aged 60 years and older stratified by age in the VHM&PP cohort

	**Women** Odds ratio (95% Confidence interval)†		**Men** Odds ratio (95% Confidence interval)†	
	Age ≤70 years	Age >70 years	Age ≤70 years	Age >70 years
**Systolic blood pressure**				
Increase of 10 mm Hg	0.94 (0.83-1.06)	0.89 (0.80-1.00)	1.08 (0.91-1.29)	0.80 (0.68-0.94)
Dichotomous (mmHg)				
<140	1.00	1.00	1.00	1.00
≥140	0.88 (0.52-1.48)	0.63 (0.41-0.97)	1.56 (0.70-3.45)	0.42 (0.23-0.78)
Categorical (mmHg)				
<120	0.72 (0.20-2.56)	1.31 (0.52-3.26)	1.20 (0.24-5.96)	3.56 (1.32-9.62)
120-<140	1.00	1.00	1.00	1.00
140-<160	0.91 (0.51-1.63)	0.67 (0.40-1.11)	1.42 (0.56-3.59)	0.61 (0.28-1.36)
160-<180	0.67 (0.30-1.48)	0.70 (0.39-1.27)	2.07 (0.75-5.69) ‡	0.55 (0.23-1.32) ‡
≥180	0.81 (0.31-2.13)	0.50 (0.21-1.20)		
**Diastolic blood pressure**				
Increase of 5 mm Hg	0.89 (0.78-1.01)	0.93 (0.84-1.03)	1.14 (0.97-1.35)	0.77 (0.65-0.91)
Dichotomous (mmHg)				
<90	1.00	1.00	1.00	1.00
≥90	0.43 (0.23-0.78)	0.80 (0.51-1.27)	1.67 (0.81-3.44)	0.48 (0.21-1.09)
Categorical (mmHg)				
<80	0.61 (0.31-1.18)	1.19 (0.72-1.98)	0.98 (0.35-2.76)	2.36 (1.20-4.64)
80-<90	1.00	1.00	1.00	1.00

90-<100	0.44 (0.22-0.87)	0.88 (0.51-1.52)	1.67 (0.78-3.58) #	0.66 (0.27-1.60) #
≥100	0.25 (0.08-0.83)	0.78 (0.35-1.70)		
**Mean arterial pressure**				
Increase of 10 mm Hg	0.84 (0.68-1.04)	0.84 (0.70-1.00)	1.22 (0.92-1.61)	0.62 (0.46-0.82)

† Model 2: adjusted for subjective feeling of illness and number of medical conditions
‡ Systolic blood pressure ≥160 mm Hg
# Diastolic blood pressure ≥90 mm Hg
