# Supplementary material for: Blood pressure and falls in community-dwelling people aged 60 years and older in the VHM&PP cohort
Source: BMC Geriatr. 2013 May 21;13:50. doi: 10.1186/1471-2318-13-50 (PMC3663706; doi:10.1186/1471-2318-13-50)
Supplement: Additional file 2: Table SB — Influence of systolic, diastolic and mean arterial blood pressure on falls in women and men aged 60 years and older stratified by the time period between BP measurement and outcome in the VHM&PP cohort. [file 1471-2318-13-50-S2.pdf]

Additional Table B: Influence of systolic, diastolic and mean arterial blood pressure on falls in women and men aged 60 years and older stratified by the time period between BP measurement and outcome in the VHM&PP cohort,

	**Women** Odds ratio (95% Confidence interval)†		**Men** Odds ratio (95% Confidence interval)†	
	Time period		Time period	
	<571 days	≥571 days	<571 days	≥571 days
**Systolic blood pressure**				
Increase of 10 mm Hg	0.91 (0.80-1.02)	0.91 (0.82-1.02)	0.88 (0.74-1.04)	0.95 (0.80-1.13)
Dichotomous (mmHg)				
<140	1.00	1.00	1.00	1.00
≥140	0.62 (0.39-0.99)	0.83 (0.51-1.33)	0.72 (0.37-1.41)	0.69 (0.34-1.41)
Categorical (mmHg)				
<120	0.53 (0.15-1.85)	0.95 (0.77-4.96)	3.08 (1.03-9.22)	1.90 (0.56-6.43)
120-<140	1.00	1.00	1.00	1.00
140-<160	0.59 (0.35-1.01)	1.00 (0.57-1.77)	1.08 (0.47-2.49)	0.66 (0.27-1.61)
160-<180	0.44 (0.22-0.91)	1.00 (0.52-1.93)	0.84 (0.32-2.21) ‡	1.03 (0.42-2.55) ‡
≥180	0.75 (0.32-1.74)	0.54 (0.20-1.50)		
**Diastolic blood pressure**				
Increase of 5 mm Hg	0.93 (0.83-1.05)	0.91 (0.81-1.01)	0.89 (0.75-1.06)	0.97 (0.81-1.16)
Dichotomous (mmHg)				
<90	1.00	1.00	1.00	1.00
≥90	0.65 (0.38-1.08)	0.60 (0.36-0.99)	0.83 (0.40-1.73)	0.98 (0.46-2.08)
Categorical (mmHg)				
<80	0.94 (0.54-1.66)	0.83 (0.47-1.47)	2.49 (1.20-5.19)	1.10 (0.45-2.68)

80-<90	1.00	1.00	1.00	1.00
90-<100	0.72 (0.40-1.29)	0.57 (0.31-1.05)	1.20 (0.53-2.74) #	1.00 (0.46-2.21) #
≥100	0.43 (0.15-1.23)	0.57 (0.25-1.29)		
**Mean arterial pressure**				
Increase of 10 mm Hg	0.85 (0.70-1.04)	0.83 (0.68-1.00)	0.79 (0.59-1.06)	0.92 (0.68-1.25)

† Model 2: adjusted for age, subjective feeling of illness and number of medical conditions
‡ Systolic blood pressure ≥160 mm Hg
# Diastolic blood pressure ≥90 mm Hg
