# Supplementary material for: Blood pressure and falls in community-dwelling people aged 60 years and older in the VHM&PP cohort
Source: BMC Geriatr. 2013 May 21;13:50. doi: 10.1186/1471-2318-13-50 (PMC3663706; doi:10.1186/1471-2318-13-50)
Supplement: Additional file 3: Table SC — Influence of systolic, diastolic and mean arterial blood pressure on falls additionally adjusted for BP treatment in women and men aged 60 years and older in the VHM&PP cohort. [file 1471-2318-13-50-S3.pdf]

Additional Table C: Influence of systolic, diastolic and mean arterial blood pressure on falls additionally adjusted for BP treatment in women and men aged 60 years and older in the VHM&PP cohort

	**Women** Odds ratio (95% Confidence interval)		**Men** Odds ratio (95% Confidence interval)	
	Model 3*	Model 4†	Model 3*	Model 4†
**Systolic blood pressure**				
Increase of 10 mm Hg	0.91 (0.84-1.00)	0.91 (0.83-1.00)	0.91 (0.80-1.03)	0.92 (0.80-1.04)
Dichotomous (mmHg)				
<140	1.00	1.00	1.00	1.00
≥140	0.74 (0.52-1.05)	0.68 (0.48-0.98)	0.75 (0.45-1.25)	0.77 (0.46-1.29)
Categorical (mmHg)				
<120	1.37 (0.66-2.84)	1.19 (0.57-2.50)	2.93 (1.29-6.65)	2.42 (1.03-5.67)
120-<140	1.00	1.00	1.00	1.00
140-<160	0.79 (0.53-1.19)	0.72 (0.48-1.09)	0.96 (0.52-1.78)	0.93 (0.50-1.73)
160-<180	0.77 (0.47-1.27)	0.69 (0.42-1.15)	0.98 (0.49-1.95)‡	0.98 (0.49-1.99)‡
≥180	0.61 (0.30-1.24)	0.62 (0.30-1.26)		
**Diastolic blood pressure**				
Increase of 5 mm Hg	0.93 (0.85-1.01)	0.93 (0.85-1.01)	0.92 (0.81-1.04)	0.92 (0.80-1.05)
Dichotomous (mmHg)				
<90	1.00	1.00	1.00	1.00
≥90	0.66 (0.45-0.96)	0.61 (0.41-0.90)	0.89 (0.52-1.53)	0.94 (0.54-1.63)
Categorical (mmHg)				
<80	0.91 (0.60-1.38)	0.88 (0.58-1.34)	1.86 (1.07-3.25)	1.80 (1.02-3.17)
80-<90	1.00	1.00	1.00	1.00

90-<100	0.68 (0.44-1.05)	0.61 (0.39-0.97)	1.09 (0.61-1.94)#	1.13 (0.62-2.04)#
≥100	0.57 (0.29-1.09)	0.51 (0.25-1.05)		
**Mean arterial pressure**				
Increase of 10 mm Hg	0.85 (0.73-0.99)	0.85 (0.73-0.99)	0.83 (0.66-1.04)	0.84 (0.67-1.05)

* Model 3: adjusted for age and BP treatment
† Model 4: adjusted for age, subjective feeling of illness, number of medical conditions (without BP treatment) and BP treatment
‡ Systolic blood pressure ≥160 mm Hg
# Diastolic blood pressure ≥90 mm Hg
